# Supplementary material for: Effects of Dietary Protein Intake on Cutaneous and Systemic Inflammation in Mice with Acute Experimental Psoriasis
Source: Nutrients. 2021 May 31;13(6):1897. doi: 10.3390/nu13061897 (PMC8228490; doi:10.3390/nu13061897)
Supplement: Supplementary file 1 [file nutrients-13-01897-s001.zip › nutrients-1225441-supplementary.pdf]

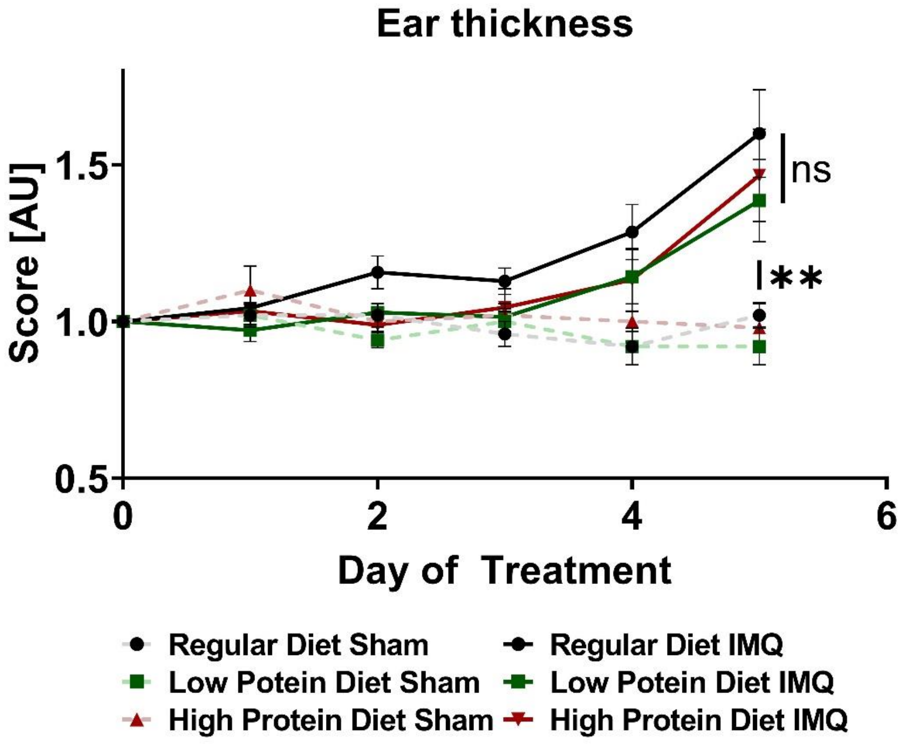

Figure S1. Modulating dietary protein does not affect skin thickness in IMQ-induced psoriasis-like skin disease. Quantification of ear thickness during sham or IMQ-treatment in the different dietary groups (n = 8–13; 2-way ANOVA).

A)

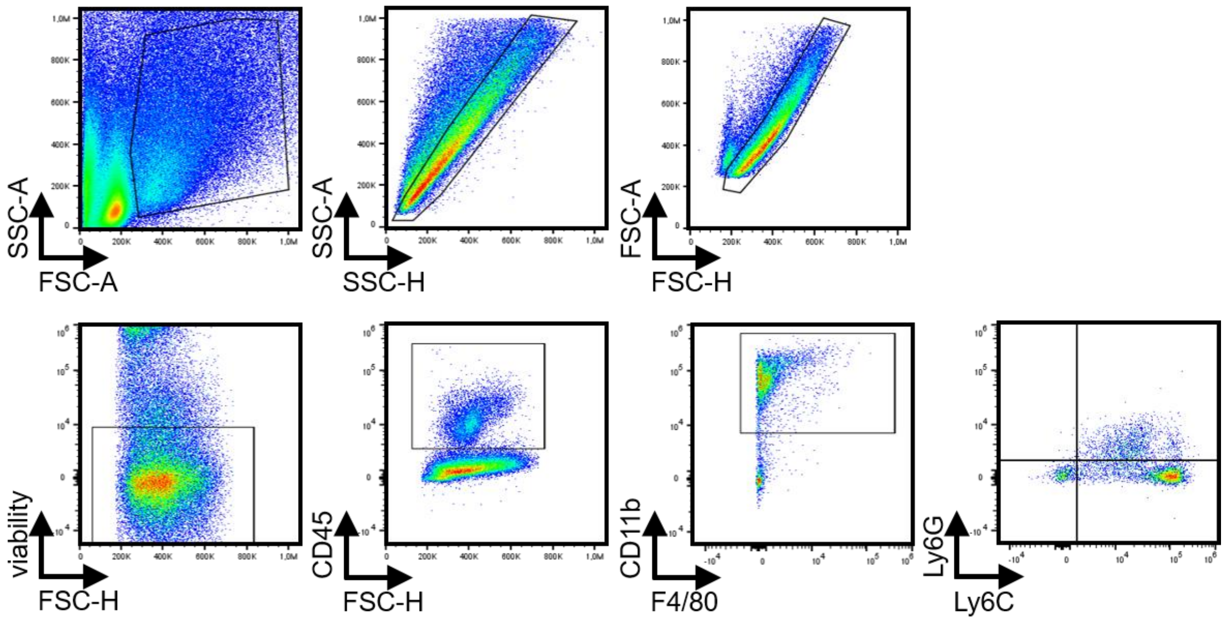

B)

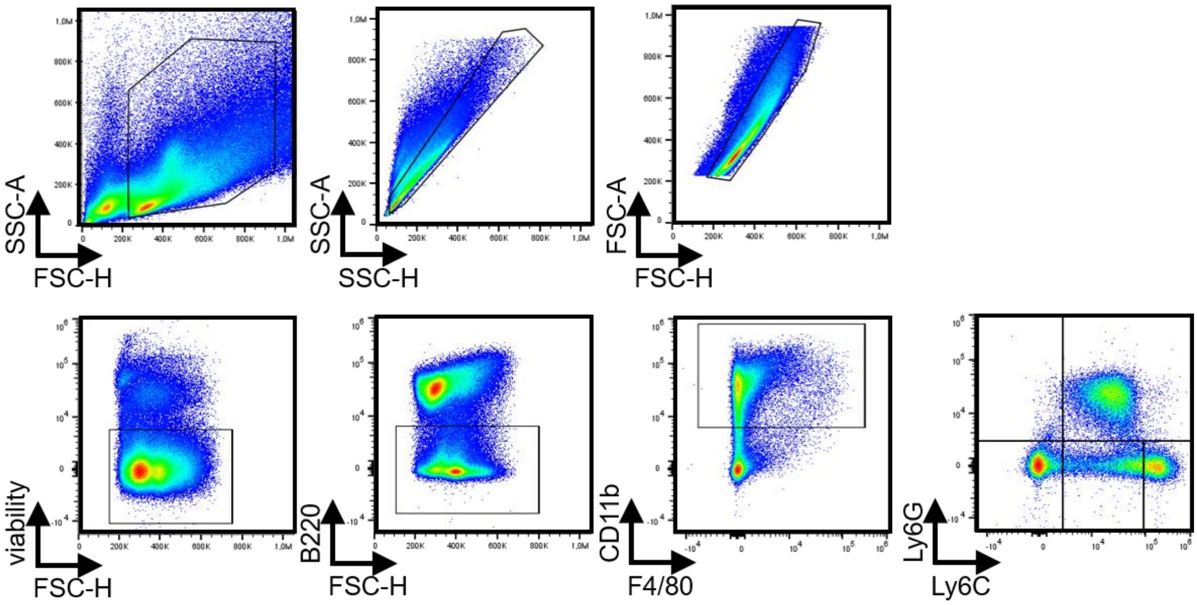

Figure S2. Gating strategy of ear (A) and spleen (B). .
